# Health Professionals’ knowledge and practice on basic life support and its predicting factors in Ethiopia: Systematic review and meta-analysis

**DOI:** 10.1371/journal.pone.0297430

**Published:** 2024-04-09

**Authors:** Worku Necho Asferie, Demewoz Kefale, Amare Kassaw, Amare Simegn Ayele, Gedefaye Nibret, Yohannes Tesfahun, Habitamu Shimels Hailemeskel, Solomon Demis, Shegaw Zeleke, Tigabu Munye Aytenew

**Affiliations:** 1 Department of pediatric and neonatal Nursing, College of Health Science, Debre Tabor University, Debre Tabor, Ethiopia; 2 Department of Midwifery, College of Health Science, Debre Tabor University, Debre Tabor, Ethiopia; 3 Department of Emergency and Critical Care Nursing, College of Health Science, Debre Tabor University, Debre Tabor, Ethiopia; 4 Department of Nursing, College of Health Science, Debre Tabor University, Debre Tabor, Ethiopia; Debre Berhan University, ETHIOPIA

## Abstract

**Background:**

Basic Life Support (BLS) is a sequence of care provided to patients who are experiencing respiratory arrest, cardiac arrest, or airway obstruction. Its main purpose is to maintain the airway, breathing, and circulation through CPR. This review aimed to estimate the pooled prevalence of Health Professionals’ knowledge and practice on basic life support in Ethiopia.

**Method:**

Eligible primary studies were accessed from international database (PubMed, Google Scholar, Hinari databases) and grey literatures found in online repositories. The required data were extracted from those studies and exported to Stata 17 for analysis. A weighted inverse-variance random-effects model and Der Simonian-Laird estimation method were used to compute the overall pooled prevalence of Health Professional’s knowledge, practice of basic life support and its predictors. Variations across the included studies were checked using forest plot, funnel plot, I^2^ statistics, and Egger’s test.

**Result:**

A total of 5,258 Health Professionals were included from 11 studies. The pooled prevalence of knowledge and practice outcomes on basic life support in Ethiopia were 47.6 (95% CI: 29.899, 65.300, I^2^: 99.21%) and 44.42 (95% CI: 16.42, 72.41, I^2^: 99.69) respectively. Educational status of the Professional’s was significantly associated with knowledge outcome. Those who had degree and above were 1.9 times (AOR: 1.90 (1.24, 2.56)) more likely knowledgeable on basic life support than under degree.

**Conclusion:**

The overall pooled estimates of Health Professionals knowledge and practice on basic life support was considerably low. The educational status of the Health Professionals was significantly associated with knowledge outcome. The Health Professionals and responsible stakeholders should focus on the basic life support at Health Institutions. The professionals should advance their knowledge and skill on basic life support for the patients.

## Background

Basic Life Support (BLS) is a sequence of care provided to patients who experiencing respiratory arrest, cardiac arrest, or airway obstruction. Its main purpose is to maintain the airway, breathing, and circulation through cardiopulmonary resuscitation (CPR) [1]. It requires knowledge and skills in CPR, using automated external defibrillators (AED) and relieving airway obstructions in patients of every age [2–4].

The BLS program is designed to deliver knowledge to a wide range of healthcare professionals about several life-threatening emergencies. It also demonstrates training to provide CPR, use an AED, and relieve choking in a safe, timely, and effective manner to treat those emergencies. It is mandatory for all healthcare professionals to have comprehensive knowledge and skill about BLS [5, 6].

Cardiopulmonary resuscitation is a series of emergency lifesaving actions which is performed in an effort to manually resuscitate a person in cardiac arrest. It is a critical component of basic life support (BLS) as the first-line response to cardiac arrest before defibrillation and advanced life support become available [7]. It helps to preserve intact brain function until further measurements are taken to restore spontaneous blood circulation and breathing in a person who is in cardiac arrest. It is recommended in those who are unresponsive with no breathing or abnormal breathing [8].

In-hospital cardiac arrest (IHCA) is a major adverse event for hospitalized patients with a reported incidence of 1.6/1000 admissions in European countries [9]. Early recognizing and intervention saves lives of the patients [10]. Adequate knowledge and skills of health care providers with regard to the manoeuvre and techniques of CPR to prevent irreversible organ damages and improves the chances of survival of cardiac arrest victims [11, 12]. But, Some studies done in Africa showed most of the Health care providers had poor knowledge and practice regarding to basic life support/resuscitation at Hospitals [13–16].

Different factors affect the knowledge and skills of Health Professionals while conducting basic life support for the patients admitted at ICU like training of the resuscitation, years of clinical experience, educational status of the professionals and frequently involving in the resuscitation activities [16–18]. There was no study conducted the pooled estimates of the related topics in Ethiopia. Therefore, this study aimed to identify the pooled estimates of Health Professionals’ knowledge and practice on basic life support and associated factors in Ethiopia.

## Methods

This systematic review and meta-analysis was carried out using a methodology of Preferred Reporting Items for Systematic Review and Meta-analysis (PRISMA) [19]. It was carried out by conducting a systematic synthesis of the pertinent primary studies on the Knowledge and practice of Health Professionals on basic life support in Ethiopia. The review protocol has been submitted for registration in an international prospective register for systematic reviews.

### Searching strategy

For explicit presentation of the reviewing questions and searching criteria we followed adapted PICO or “PEO” that (Population, Exposure and Outcomes) for creating the MeSH Terms to retrieve the potential studies in the database inclusion. Based on this;

- **Population**: Health Care Providers, Health Professionals.
- **Exposure:** basic life support/resuscitation
- **Outcome**: Knowledge and Practice of Health Professionals’ on basic life support

**Study Design:** Observational studies and

**Setting**: Ethiopia

**Our Research question** “What is the national prevalence of Health Professional’s knowledge and practice on basic life support in Ethiopia?”

Two approaches were followed to search potentially relevant studies. The electronic database search (PubMed, Google Scholar, Hinari, and Institution research repositories) and the manually search of the reference list of the previous prevalence studies were carried out to retrieve more articles. “Health Professionals”, “Health Care Providers”, “Knowledge”, “Skill”, “Practice”, “Resuscitation”, “Cardiopulmonary Resuscitation”, “Basic life Support” and “Ethiopia” were the key terms mostly used for retrieving reputable articles from database both using separation and in-combination with the balloon operators like “OR” or “AND”, Truncations(….*) and Phrase (….). The articles were searched from 01/01/2023 to 8/20/2023. Finally, all studies which were in agreement with the review question were retrieved and screened for inclusion criteria

### Eligibility

#### Inclusion and exclusion criteria

Primary studies of any design that described the prevalence, proportion, and extent of knowledge and practice of basic life support among Health Professionals were included in this analysis. But, primary studies were excluded for any of the following reasons: (a) there was no information on the prevalence of knowledge and practice outcome; (b) there was no full text; (c) there was a low quality score; (d) the full text of the article was not accessible after 3 emails were sent to the corresponding author; and (e) there were no narrative reviews, editorials, correspondence, abstracts, or methodological studies.

All retrieved studies were independently evaluated for eligibility by two authors (W. A and T. A), and any disagreements or inconsistencies were settled by the involvement of a third author (A.K), who broke the disagreement.

### Outcome variable measurements

The knowledge and practice of basic life support (defined here as knowledge of resuscitation, knowledge of cardiopulmonary resuscitation, knowledge of basic life support and practice of resuscitation, cardiopulmonary resuscitation and basic life support) among Health professionals was the main outcome variable. Those Health care professionals who score 70% and above for the knowledge measuring items were considered as Good knowledge otherwise poor knowledge [20, 21]. Those Health care providers who score 60% of the practice measuring items of basic life support would considered having adequate practice [22]. The second outcome was to assess factors that affect the pooled knowledge and practice on basic life support. The association of outcome and factors was examined using odd ration that was calculated by two by table (or = ad/bc).

### Study screening and selection

Search results were first downloaded into Endnote version 7 and duplicates were removed. Then, selection of studies was conducted in 2 stages. First, title and abstract screening was done. Then, full-text reviewing was conducted. Through title and abstract screening by 2 independent authors (WA and TA), studies that mentioned the prevalence /magnitude/proportion knowledge and practice on basic life support among Health Professionals were selected for full text review. Then, from full-text reviewing, any article classified as potentially eligible by either author was considered as a full text and screened by both authors independently. At times of disagreement where a consensus could not be reached between the authors, a third author (AK) reviewed and resolved the disagreements

### Critical appraisal and reliability checkup

After screening the relevant studies, selected studies were critically appraised for methodological validity using Joanna Briggs Institute (JBI) appraisal tool for prevalence studies [23]. The tool had a total of 9 questions (Q1-Q9) and those studies with positive answer of more than 50% of the tool (i.e. ‘Yes’ for 5 or more question of JBI tool) were included for this meta-analysis **(Table 1).**The risk of bias for each primary studies would evaluate using the adopted tool from hoy et al. [24] **(Table 2).** The scoring was done by 2 investigators (SD and DK) with the discrepancies were resolved with discussion and consensus. When the disagreement between the 2 authors was not resolved with discussion, the third author (AA) was involved as a breaker. During critical appraisal of each primary study, more emphasis was given to the appropriateness of the study objectives, study design, sampling technique, data collection technique, statistical analysis, any sources of bias and its management technique.

**Table 1:** Quality assessment of the included studies using the Joanna Briggs Institute (JBI) quality appraisal criteria, Ethiopia, 2023.

Quality Appraisal for included studies in this systematic review and meta-analysis in Ethiopia, 2023											
S/N	Author (Year)	Criteria								Scores	Overall quality
		Clearly defined inclusion criteria	Describing the study settings participants	Valid &reliable exposure measurement	Objective &standard criteria for measurement	Identified confounder	Strategies to deal with confounder	Valid & reliable outcome measurement	Appropriate statistical analysis		
1.	Kelkay et al. 2018 [35]	N	Y	Y	Y	N	N	Y	Y	5	Low risk
2.	Mersha. A et al.,2020 [39]	Y	Y	Y	Y	N	Y	Y	Y	7	Low risk
3.	Mersha AT et al., 2020 [18]	N	Y	Y	Y	N	Y	Y	Y	6	Low risk
4.	(Abebe et al.2021) [37]	Y	Y	Y	Y	N	Y	Y	Y	7	Low risk
5.	Bizuwork K et al, 2019 [36]	N	Y	Y	Y	N	Y	Y	Y	6	Low risk
6.	Bikamo .E, 2021 [40]	Y	Y	Y	Y	N	Y	Y	Y	7	Low risk
7.	Benwu.MK, 2021[42]	N	Y	Y	Y	N	Y	Y	Y	6	Low risk
8.	Abebaw M et al… 2022 [41]	Y	Y	Y	Y	N	Y	Y	Y	7	Low risk
9.	Bekele et al, 2021[38]	Y	Y	Y	Y	N	Y	Y	Y	7	Low risk
10.	Sintayehu et al,2020 [43]	Y	Y	Y	Y	N	Y	Y	Y	7	Low risk
11.	Getuta, 2022 [17]	Y	Y	Y	Y	N	Y	Y	Y	7	Low risk

Note: Y- Yes, N- No

**Table 2:** Risk of bias assessment of the included studies.

S/N	Author (Year)	Criteria										Scores	Overall risk of bias
		External validity				Internal validity							
		Q1	Q2	Q3	Q4	Q5	Q6	Q7	Q8	Q9	Q10		
1.	Kelkay et al. 2018	N	Y	Y	Y	Y	N	Y	Y	Y	Y	8	Low risk
2.	Mersha. A et al.,2020	N	Y	Y	Y	Y	Y	Y	Y	N	Y	8	Low risk
3.	Mersha AT et al., 2020	N	Y	Y	Y	Y	Y	Y	Y	N	Y	8	Low risk
4.	(Abebe et al.2021)	N	Y	N	Y	Y	Y	Y	Y	Y	Y	8	Low risk
5.	Bizuwork K et al, 2019	N	Y	Y	Y	Y	Y	Y	Y	N	Y	8	Low risk
6.	Bikamo .E, 2021	N	Y	Y	Y	N	Y	Y	Y	Y	Y	8	Low risk
7.	Benwu.MK, 2021	N	Y	Y	Y	Y	N	Y	Y	Y	Y	8	Low risk
8.	Abebaw M et al, 2022	N	Y	Y	Y	Y	Y	Y	Y	N	Y	8	Low risk
9.	Bekele et al, 2021	N	Y	Y	Y	N	Y	Y	Y	Y	Y	8	Low risk
10.	Sintayehu et al,2020	N	Y	Y	Y	Y	Y	Y	Y	N	Y	8	Low risk
11.	Getuta, 2022	N	Y	Y	Y	Y	Y	Y	Y	Y	N	8	Low risk

**Note:** Y: yes, N: No

### Data extraction

The investigators extracted the required data using a pre-tested data extraction format using Microsoft excel 2010. The following information was taken from the studies: the first author, the region where the study was conducted, specific study area, study design, study publication year, study sample size, response rate of the study, and the prevalence of knowledge and practice of basic life support. Additionally, variables which were significantly associated with each primary studies extracted considering the following points: adjusted odd ration and their confidence interval to computed their Standard Errors and log odd ration that used for the final analysis to pooled signicant variables for the reviewed question. Any discrepancies between the two authors regarding the data extraction process were resolved through discussion and agreement. Involving a third reviewer also helped to address the variation.

### Stastical analysis

The necessary data were extracted using Microsoft excel 2010 and exported to Stata 17 (STATA Corporation, College Station Texas) for analysis. The primary study was summarized using table and forest plot. The Authors calculated the standard errors of the prevalence of knowledge and practice for each original articles using binomial formula. We check the level of Heterogeneity among the reported prevalence of the studies using the Cochrane Q^**2**^ and I^**2**^ stastics [25, 26]. The heterogeneity was quantified high (considerable), moderate, low as 75% and more, 50–75% and 25% and less respectively. The random effects model was used to estimate the der Simonian and Laird’s pooled effects since test statics showed there was a considerable heterogeneity among studies (I^**2**^ = 99.21, P = 0.000 for knowledge and I^**2**^ = 99.69, P = 0.0019 for practice). The publication bias was conducted using subjectively by funnel plot and objectively using egger’s test with 5% significant level. In egger’s test p-value less 0.05 indicates the presence of publication bias while greater than 0.05 indicates the absence of bias [27, 28]. If publication bias is noticed in the Random effects model, the estimate is determined by using Duval and Tweedie’s trim and Fill analysis. In addition, subgroup analysis was done using region of studies in order to reduce the random heterogeneity between the estimates of the primary studies.

## Result

### Literature searching findings

**Fig 1:**
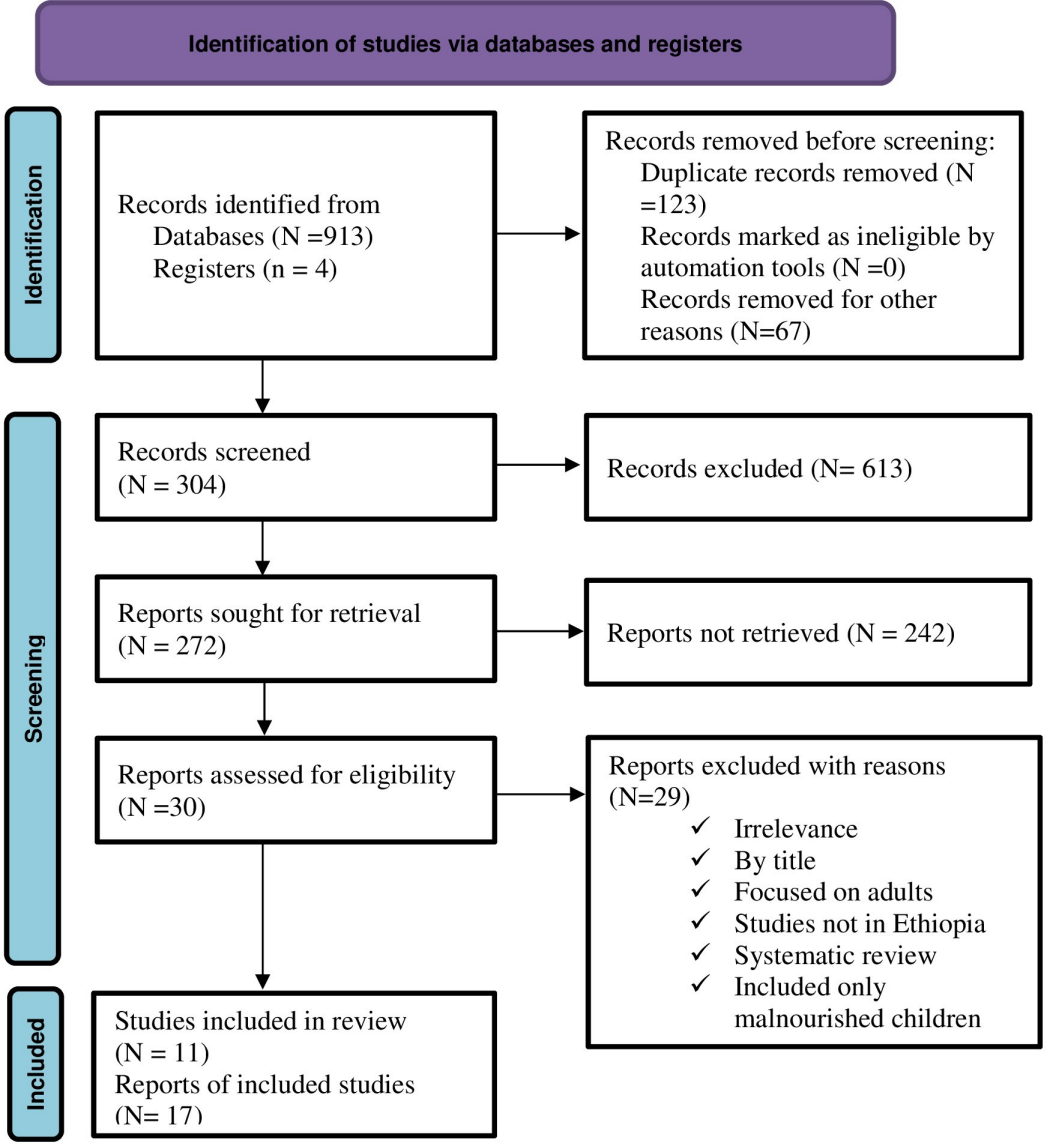
PRISMA flow diagram of article selection for systematic review and meta-analysis of the Health Professionals’ knowledge and practice on basic life support and its predicting factors in Ethiopia.

A total of 917 articles were retrieved after a thorough search of both published and unpublished sources. Out of 917 articles, 913 were collected from databases. The remaining four articles were obtained from institutional research repository at Addis Abeba University. Of the 913 articles found through database searching, 223 were found through Google Scholar, 325 through PubMed, and 365 through Hinari. About 857 articles were eliminated due to duplication, different countries and different interest question. Additionally, 6 articles [29–34] were excluded due to target group and methodological differences. Finally, 11 articles were included for this systematic review and meta-analysis **(Fig 1)**.

### Characteristics of the original studies

The characteristics of 11 studies included in this review have been described in details in Table 3. All studies were used cross sectional study design with sample size ranges of 143, in Amhara regional state and 445 in SNNP. These studies were conducted from 2014 to 2022. In this meta-analysis, a total 3849 sample size was used to estimate the overall prevalence of Health professional’s Knowledge and practice of resuscitation in Ethiopia. The 11 studies were conducted at different regions of Ethiopia; Amhara region 5 studies [18, 35–38], 1 South Nation Nationality and People (SNNP) [39], 2 in Addis Ababa [40, 41], 1 in Harar [14], and 1 in Tigray [42].

**Table 3:** Descriptive summary of 11 studies reporting the knowledge, practice and associated factors of resuscitation/basic life support among Health Professionals in Ethiopia included in the systematic review and meta-analysis, 2023.

Author (year)	region	Publication Year	Sample size	response rate%	Quality score	Prevalence (95% CI)	Data Collection Techniques	Fund
Kelkay et al. 2018 [35]	Amhara	2018	397	97.7	5	38.60 (33.81, 43.39)	Self-administered	UOG
Mersha. A et al.,2020 [39]	SNNPR	2020	445	96.4	7	76.2 (72.24, 80.16)	Self-administered	AU
Mersha AT et al., 2020 [18]	Amhara	2020	424	95.7	6	25.1(20.97, 29.23)	Self-administered	Not reported
(Abebe et al.2021) [37]	Amhara	2021	352	92	7	22.2(17.86, 26.54)	Self-administered	Not reported
Bizuwork K et al, 2019 [36]	Amhara	2019	143	100	6	32.9(25.20, 40.60)	Self-administered	Not Funded
Bikamo .E, 2021 [40]	Addis Ababa		215	96.5	7	41.5(34.91, 48.09)	Interview	Not reported
Benwu.MK, 2021[42]	Tigray	2021	245	100	6	57.55(51.36, 63.74)	Interview	Not Funded
Abebaw M et al… 2022 [41]	Addis Ababa	2022	409	100	7	87.3(84.07, 90.53)	Self-administered	Not funded
Bekele et al, 2021 [38]	Amhara	2021	360	100	7	46.7(41.55, 51.85)	Self-administered	UOG
Sintayehu et al,2020 [43]	Harar	2020	437	97.7	7	11.2(8.24, 14.16)	Oberservation	Not reported
Getuta, 2022 [17]	SNNP	2020	422	100	7	31.8(27.36, 36.24)	Interview	Not reported

### Risk of bias assessment

The tool developed by Hoy *et al* was used to assess the risk of bias for each included study [24]. The tool consists of ten items that assess four areas of bias; internal validity and external validity. Items 1–4 evaluate selection bias, non-response bias and external validity. Items 5–10 assess measure bias, analysis-related bias and internal validity. Accordingly, of the total of the twenty-six included studies, twenty-two studies scored eight of ten questions and the four studies also scored seven of ten questions. Studies were classified as ʺlow riskʺ if eight and above of ten questions received a ʺYesʺ, as ʺmoderate riskʺ if six to seven of ten questions received a ʺYesʺ and as ʺhigh riskʺ if five or lower of ten questions received a ʺYesʺ. Therefore, all included studies had low risk of bias **(Table 2).**

## Meta-analysis

### Pooled prevalence of Health Professionals’ knowledge on basic life support/resuscitation

**Fig 2:**
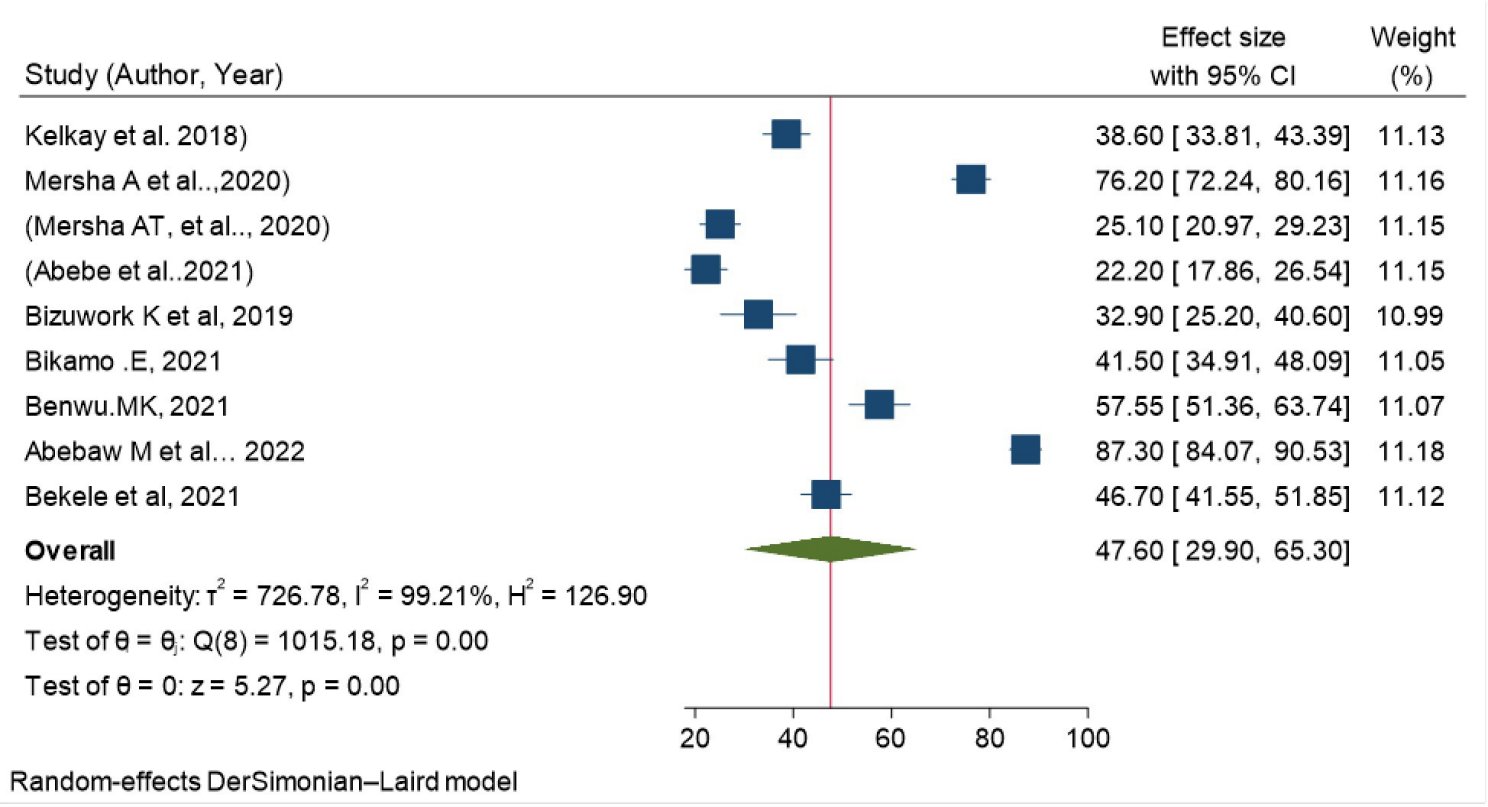
The pooled prevalence of Health Professional’s knowledge on basic life support / resuscitation in Ethiopia, 2023.

Before meta-analyzing the effect sizes of included studies, the presence of statistical variability between the included studies was checked using both visual inspection of forest plot and statistical test of variation. There was high/considerable heterogeneity among the included studies in pooled prevalence of Health Professionals’ knowledge regarding to resuscitation. The Stata generated statistical test of variation (I squared statistics = 99.21% and Chi-squared = 1015.18 (d.f = 8); P < .001) indicating high heterogeneity. Therefore, random effects model was used to estimate the pooled prevalence of health professionals’ knowledge on resuscitation in Ethiopia. The overall pooled knowledge prevalence was 47.6 (95% CI: 29.899, 65.300) **(Table 4 and Fig 2).**

**Table 4:** Pooled prevalence of Health Professionals’ knowledge on basic life support/resuscitation in Ethiopia, 2023.

Study	Effect size	95% CI	% weight
Kelkay et al. 2018	38.60	33.811, 43.389	11.13
Mersha A et al..,2020	76.20	72.243, 80.157	11.16
Mersha AT, et al.., 2020	25.10	20.973, 29.227	11.15
Abebe et al..2021	22.20	17.858, 26.542	11.15
Bizuwork K et al, 2019	32.90	25.199, 40.601	10.99
Bikamo .E, 2021	41.50	34.914, 48.086	11.05
Benwu.MK, 2021	57.55	51.361, 63.739	11.07
Abebaw M et al… 2022	87.30	84.073, 90.527	11.18
Bekele et al, 2021	46.70	41.546, 51.854	11.12
Theta(Overall)	47.60	29.899, 65.300	

Heterogeneity: tau2 = 726.7778, I2 (%) = 99.21, H^2^ = 126.90

Test of theta = 0: z = 5.27, Prob > |z| = 0.0000

Test of homogeneity: chi-square: 1015.18 with (d.f: 8), Prob > Q = 0.0000

### Investigation of heterogeneity

**Fig 3:**
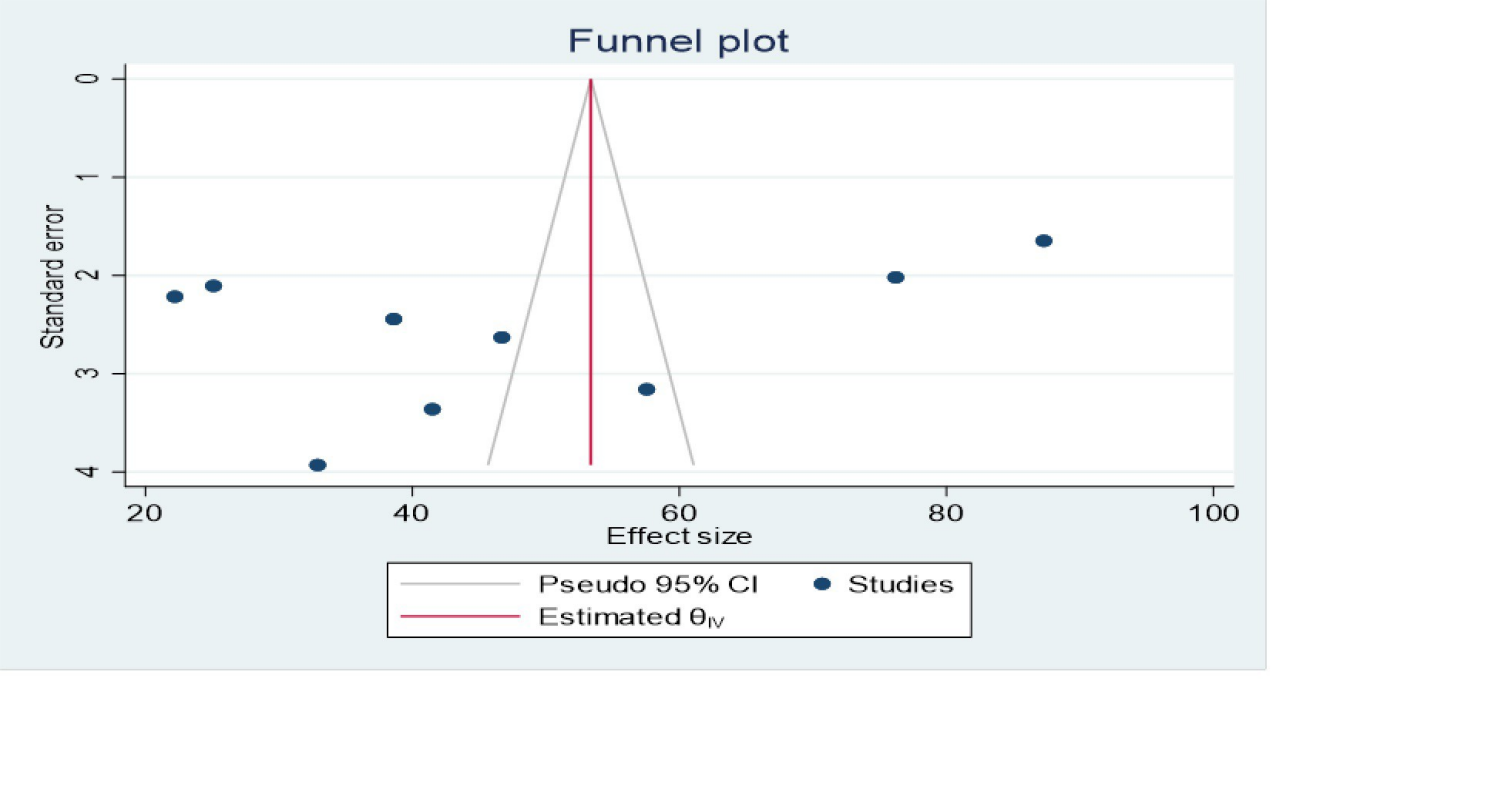
The funnel plot of among 9 studies included in this systematic review and meta-analysis.

Given the statistical heterogeneity of prevalence of knowledge of resuscitation outcome between the included primary studies (I^**2**^ statistics = 99.21%), we performed subgroup analyses based on the following criteria: (a) the subgroup analysis hypothesis was pre-specified (a priori) in the review protocol under consideration in PROSPERO; (b) there were large subgroup effect sizes; (c) Consistent interaction across the effect sizes (Prevalence) of knowledge outcome; and (d) the sub-grouping factors (region of study, and sample size were characteristics of interest measured at baseline across the studies. All the aforementioned criteria enabled us to place high confidence on the results of our subgroup analyses. Additionally, to explore the source of heterogeneity meta-funnel plot and egger stastical test were computed. The egger test revealed the absence of publication bias between the primary studies (P = 1.00). But, funnel plot also showed that there is slightly non-symmetrical distribution among primary studies **(Fig 3).**

### Subgroup analysis

**Fig 4:**
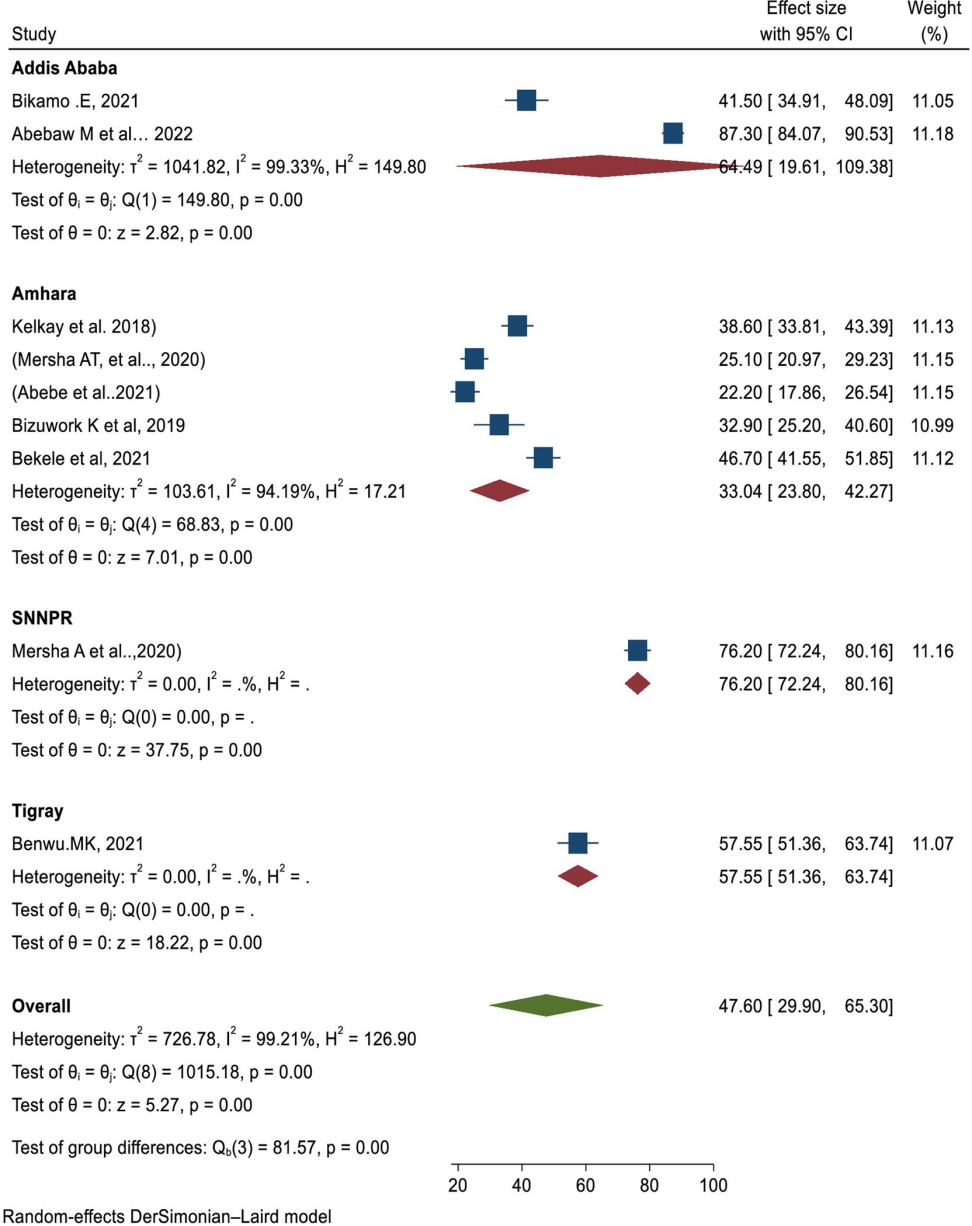
The regional subgroup analysis of Health Professionals knowledge on basic life support /resuscitation in Ethiopia, 2023.

The subgroup analysis based on region: From this analysis the highest pooled estimates of prevalence of Health Professional’s knowledge on resuscitation was seen in SSNP 76.2(95% CI: 72.24, 80.16). Whereas the lowest pooled estimate was seen in Amhara region 33.04 (95% CI: 223.80, 42.27) **(Fig 4).**

### Sensitivity analysis for knowledge prevalence

**Fig 5:**
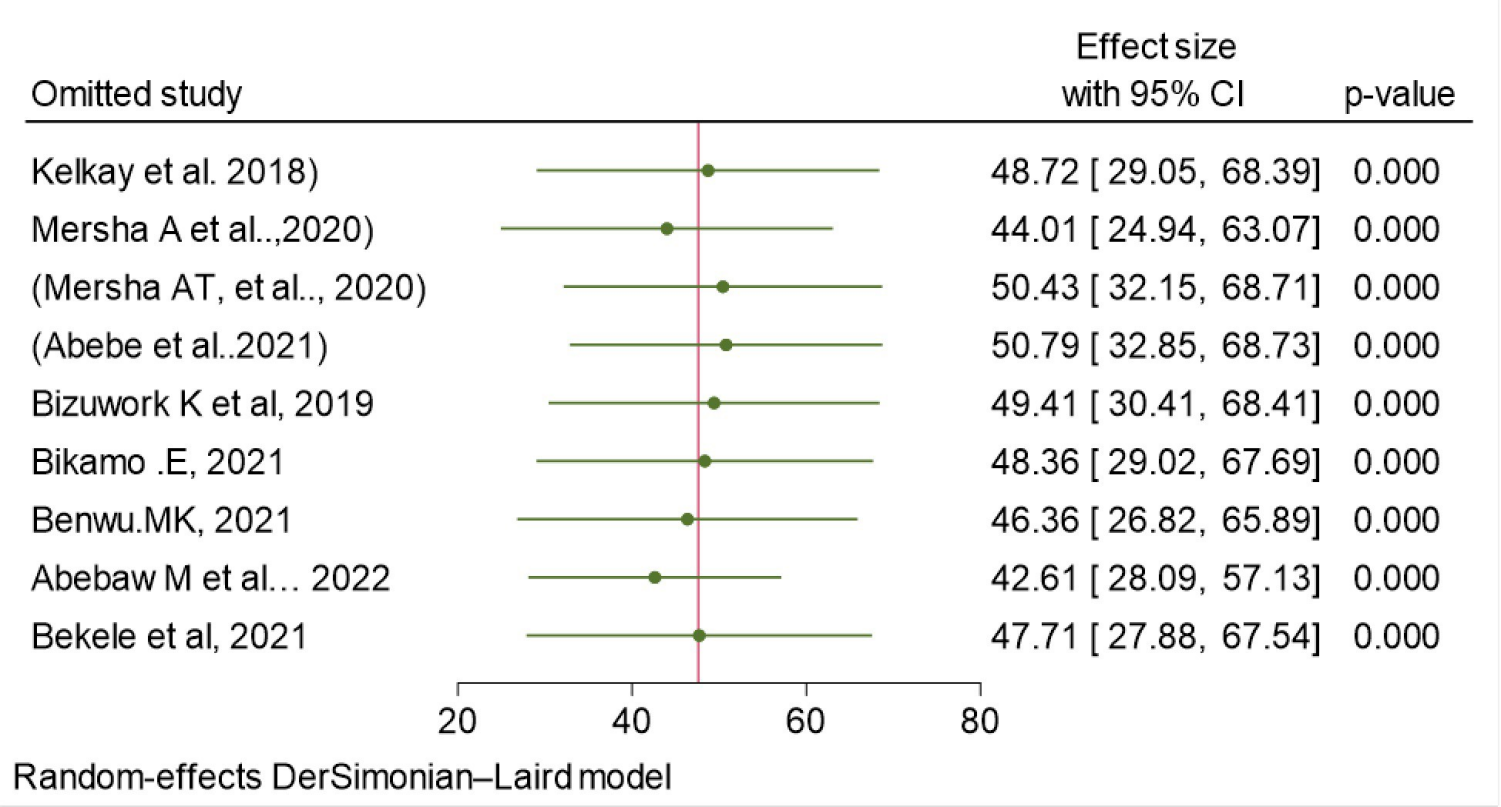
Sensitivity analysis of the pooled prevalence of Health Professionals’ knowledge on basic life support/resuscitation in Ethiopia, 2023.

Sensitivity analysis was done to identify the potential influence of single study on the pooled estimate of knowledge outcome. Using the random effects model, the result of sensitivity analysis suggested that the omission of 3 studies (Mersha AT et al, Abebe et al, and Abebaw et al) influenced the pooled estimate significantly. From the sensitivity result omission of Mersha AT et al increased the pooled estimate to 50.43 (32.15, 68.71) and the omission of Abebe et al also increased the pooled estimate to 50.79(32.85, 68.73). While, the omission of Abebaw et al was decreased the pooled estimate of knowledge outcome to 42.61(28.09, 57.13) **(Table 5 and Fig 5).**

**Table 5:** The sensitivity analysis of the pooled prevalence Health Professional’s knowledge on basic life support/ resuscitation, 2023, Ethiopia.

Omitted study	Effect size	95% CI	P-value
Kelkay et al. 2018	48.723	29.052, 68.393	0.000
Mersha A et al..,2020	44.006	24.943, 63.069	0.000
Mersha AT, et al.., 2020	50.427	32.149, 68.705	0.000
Abebe et al..2021	50.791	32.850, 68.733	0.000
Bizuwork K et al, 2019	49.414	30.414, 68.414	0.000
Bikamo .E, 2021	48.355	29.021, 67.689	0.000
Benwu.MK, 2021	46.357	26.822, 65.893	0.000
Abebaw M et al… 2022	42.607	28.089, 57.126	0.000
Bekele et al, 2021	47.708	27.876, 67.539	0.000
Combined	47.600	29.899, 65.300	0.000

### The pooled Health Professionals’ practice on basic life support/resuscitation in Ethiopia

**Fig 6:**
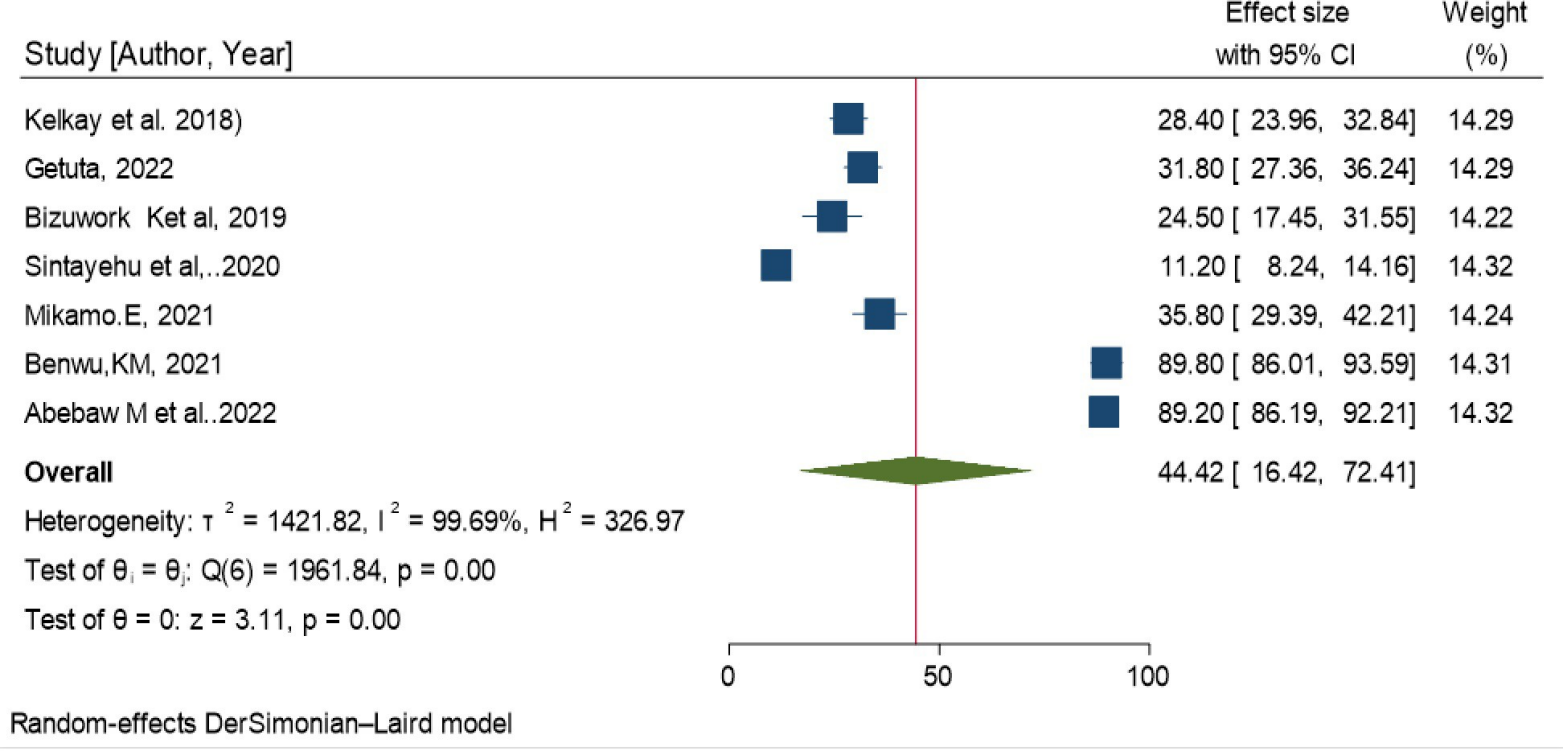
The pooled Health Professional’s practice on basic life support/resuscitation in Ethiopia, 2023.

**Fig 7:**
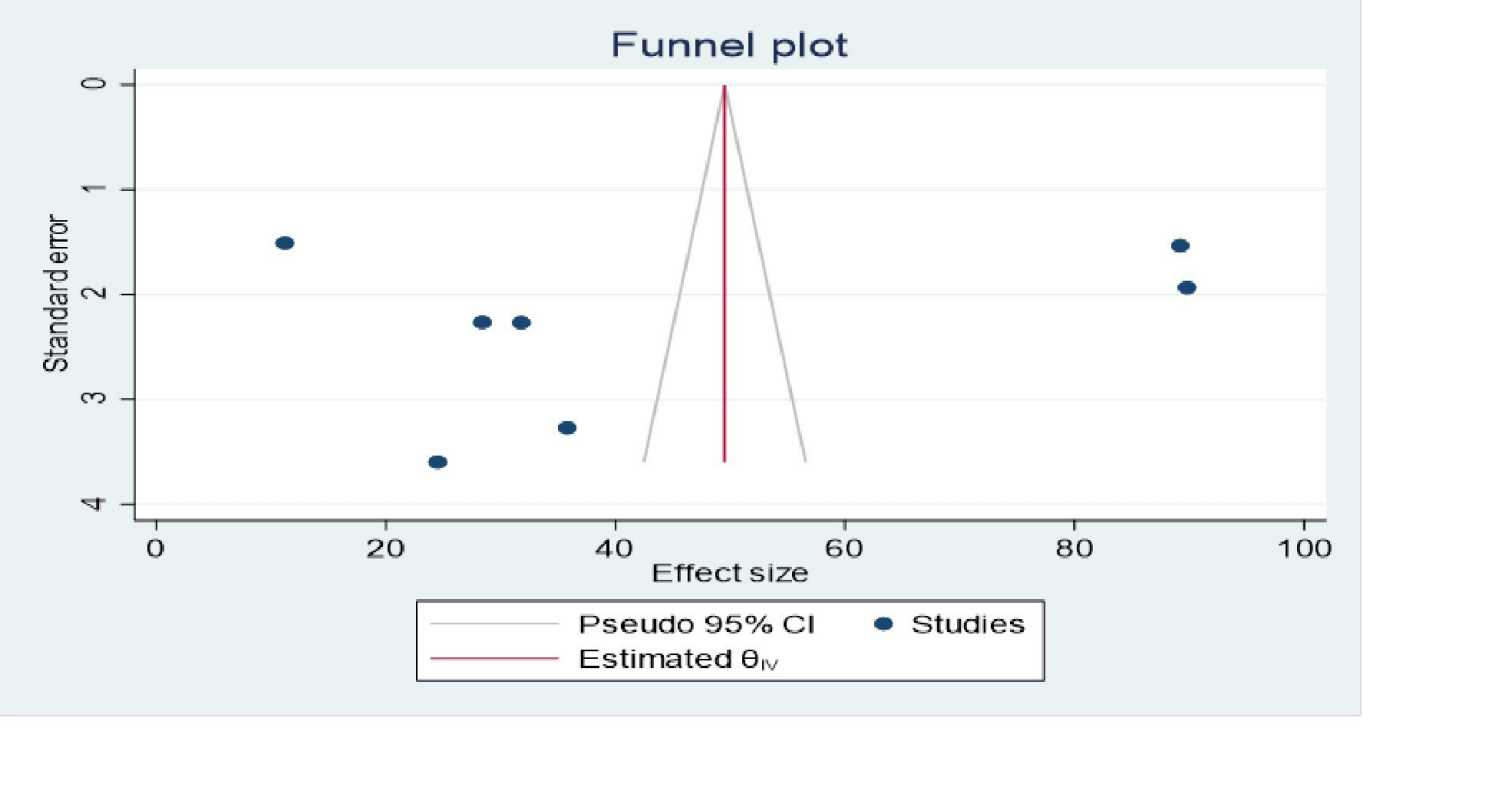
The funnel plot for Health Professional’s practice on basic life support/resuscitation in Ethiopia, 2023.

In this meta-analysis, the random effects model was used to estimate the pooled prevalence of resuscitation practice among Health Professionals. The overall pooled estimate of resuscitation practice among Health Professional’s in Ethiopia was 44.42 (95% CI: 16.42, 72.41). Nevertheless, a considerable Heterogeneity among included studies was seen and objectively detected by I^2^ stastics (**I**^**2**^ = 99.69, P-value = 0.002**) (Table 6 and Fig 6).** Additionally, to explore the source of heterogeneity meta-funnel plot and egger stastical test were computed. The egger test revealed the absence of publication bias between the primary studies (P = 0.466). But, funnel plot also showed that there is slightly non-symmetrical distribution among primary studies **(Fig 7).**

**Table 6:** The pooled estimate of Health Professionals’ practice on basic life support/ resuscitation in Ethiopia, 20203.

Study	Effect size	(95% CI)	% weight
Kelkay et al. 2018	28.40	23.96, 32.84	14.29
Getuta, 2022	31.80	27.36, 36.24	14.29
Bizuwork K . . .et al, 2019	24.50	17.45, 31.55	14.22
Sintayehu et al,2020	11.20	8.24, 14.16	14.32
Bikamo.E, 2021	35.80	29.39, 42.21	14.24
Benwu,KM, 2021	89.80	86.01, 93.59	14.31
Abebaw M et al . . .2022	89.20	86.19, 92.21	14.32
Overall	44.42	16.42, 72.41	

### Sensitivity analysis

**Fig 8:**
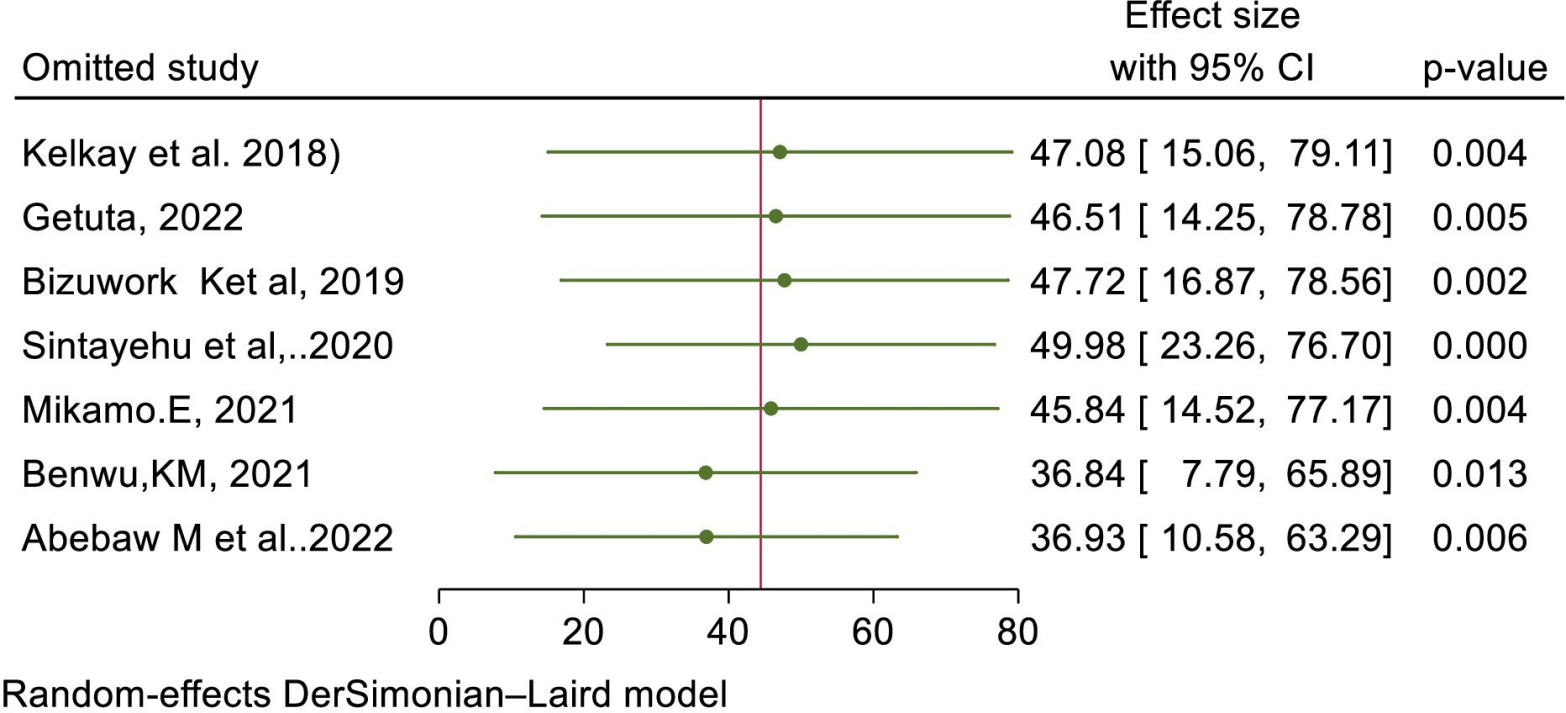
The sensitivity of the pooled prevalence of Health Professional’s practice regarding basic life support/resuscitation in Ethiopia, 2023.

Sensitivity analysis was done to identify the potential influence of single study on the pooled estimate of Practice outcome. Using the random effects model, the result of sensitivity analysis suggested that the omission of 3 studies (Sintayehu et al. 2020, Benwu, KM. 2021, Abebaw M, et. al.2022) influenced the pooled estimate significantly. From the sensitivity result omission of Sintayehu et al. 2020 increased the pooled estimate practice to 49.98 (CI: 23.26, 76.70). While, the omission of Benwu, KM. 2021 and Abebaw M, et. al. 2022 was decreased the pooled estimate of Practice outcome to 36.84(7.79, 65.89) and 36.93(10.58, 63.29) (**Table 7 and Fig 8).**

**Table 7:** Sensitivity analysis of the pooled seven primary studies about prevalence of Health Professional’s practice regarding basic life support in Ethiopia.

Omitted study	Effect size	(95% CI)	P-Value
Kelkay et al. 2018	47.08	15.06, 79.11	0.004
Getuta, 2022	46.52	14.25, 78.78	0.005
Bizuwork K et al, 2019	47.72	16.87, 78.56	0.002
Sintayehu et al,2020	49.98	23.26, 76.70	0.000
Bikamo.E, 2021	45.84	14.52, 77.17	0.004
Benwu,KM, 2021	36.84	7.79, 65.89	0.013
Abebaw M et al.2022	36.93	10.58, 63.29	0.006
Combined	44.42	16.42, 72.41	0.002

### Factors associated with Health Professional’s knowledge on basic life support/resuscitation in Ethiopia

**Fig 9:**
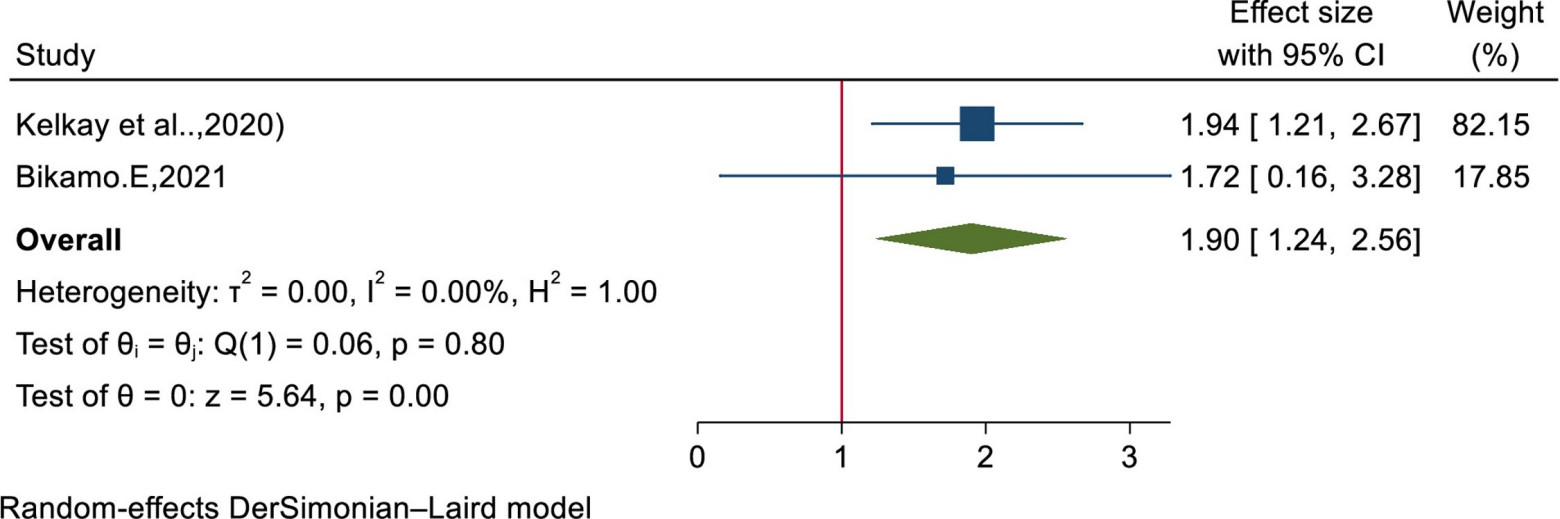
Forest plot for the association of educational status and professional’s knowledge on basic life support in Ethiopia, 2023.

Out of the included studies two studies (Kelkay et al..,2020, Mersha AT, et al.., 2020) explained that Health Professionals who had 5–10 yeas and above were knowledgeable regarding to basic life support/resuscitation. But, the pooled meta-analysis result, AOR: 1.781(0.846, 2.716), showed the variable clinical experience has no an association with the knowledge of Health care providers regarding to resuscitation. However, the pooled meta-analysis result, AOR: 1.90 (1.24, 2.56) revealed that the educational status the professionals have a significant association with their knowledge of resuscitation **(Fig 9).**

Another four primary studies explained that the knowledge of health professional were associated with recently involving in resuscitation practice. But, the pooled meta-analysis result revealed that recently involving in the resuscitation activity has no effects on the professional’s knowledge on basic life support/resuscitation. Since, the confidence intervals of the odds ratio AOR (1.43 (0.70, 2.15)) include one the recently involving in the resuscitation activity is not significantly associated with the knowledge of Health professionals.

Additionally, six primary studies were revealed the training regarding to the basic life support was significantly associated with Health Professionals knowledge of resuscitation. But, pooled analysis AOR: 0.92 (0.11, 1.72) showed that training has no association with Professionals knowledge of basic life support.

### Factors associated with the Health Professionals practice of basic life support/resuscitation in Ethiopia, 2023

The random effects model was used to identify factors that affect the pooled estimated practice of Health Professional’s in Ethiopia. Some of the primary studies explained that educational status, recently involving in resuscitation activities and taking resuscitation training had significant association with practice outcome. But, the pooled estimated analysis revealed that Knowledge of participants AOR (0.99(0.48, 1.50)), taking resuscitation training AOR (1.06(0.65, 1.46)), experience AOR (0.91(0.4, 1.42)), educational status AOR (1.23(0.34, 4.49)) were not significantly associated with practice outcome.

## Discussion

The pooled prevalence of Health Professional’s knowledge about basic life support in Ethiopia was 47.6 (95% CI: 29.90, 65.30). This finding is similar with study conducted at Pakistan(41.7%) [44], Yemen (53.6%) [45], Nigeria 43.6% [46] and Botswana 48% [47]. While this finding is lower than study conducted at India 64% [48], Islamic Republic of Iran 69% [49]. This discrepancy might due to stastical analysis (ANOVA, chi-square test), and geographical difference. Additionally, our finding was higher than study conducted at Egypt 33.92% [50].

In this study, pooled prevalence of Health professional’s practice on Basic life support in Ethiopia was 44.42 (16.42, 72.41), this finding is in line with a study conducted at Uganda 46% [51]. But, this finding was lower than a study conducted at India 66% [48] and Nigeria 65.2% [52]. The difference might due to methodological difference, d study setting.

According to the final pooled analysis, educational status of the participant’s was significantly associated with their knowledge of basic life support. Those professionals with degree and above was 1.9 time (AOR: 1.90(1.24, 2.56)) more likely knowledgeable regarding basic life support than under degree in educational status. This might be as educational level increase the Health professional’s exposure to different procedure also increased.

Generally, this meta-analysis finding revealed that the Health professional’s knowledge and practice on basic life support was poor. So, the concerned bodies should focus to enhance the professional’s capacity regarding on the implementation of basic life support at Health facilities in Ethiopia.

### Limitation

This systematic review and meta-analysis had highly considerable heterogeneity this might affect pooled estimate of the outcome. Similarly, there was small size in the included studies this also had effects on the pooled generalization. Additionally, all the included studies were also used cross sectional study design which might affects the representativeness of the pooled estimates for the target populations.

### Strength of the review

This systematic review is conducted for the first time in Ethiopia. This systematic review includes team members from different disciplines and conduct sensitivity analysis, subgroup analysis to dissolve heterogeneity among included studies. Additionally, this review also tried to incorporate all studies regarding to the research question. The risk assessments and quality assessment for each study also conducted.

## Conclusion

In conclusion, the overall pooled prevalence of Health professional’s knowledge and practice on basic life support in Ethiopia was 47.6% and 44.42% respectively. This finding was very poor to save the life of critically ill patients at emergency site. Only the educational status of the professional’s was significantly associated with knowledge outcomes. Moreover, Health Professionals and responsible stakeholders should give focus and advance on the knowledge and practice of basic life support/CPR in Ethiopia.

## Supporting information

S1 Checklist PRISMA 2020 Checklist. (DOCX)

S1 Data (XLSX)

S2 Data (XLSX)

## Abbreviation

AEP: automated external defibrillators
AOR: Adjusted odds ratio
BLS: Basic life support
CPR: Cardiopulmonary Resuscitation
JBI: Joanna Briggs Institute
IHCA: In-hospital cardiac arrest
ICU: Intensive care unit
PRISMA: Preferred reporting for items in systematic review and meta-analysis.

## References

[1] Al Enizi B.A., et al., Knowledge and attitudes about basic life support among secondary school teachers in Al-Qassim, Saudi Arabia. International journal of health sciences, 2016. 10(3): p. 415. 27610065 PMC5003585

[2] American Red Cros trianing service. Basic life support.

[3] Roshana S., et al., Basic life support: knowledge and attitude of medical/paramedical professionals. World journal of emergency medicine, 2012. 3(2): p. 141. doi: 10.5847/wjem.j.issn.1920-8642.2012.02.011 25215053 PMC4129799

[4] Chaudhary G.P., et al., Knowledge regarding Basic Life Support among Health Care Workers of the Hospital of Nepal. 2023. 2023: p. 9936114. 10.1155/2023/9936114 PMC9836805 36644299

[5] Murray C.J., et al., Disability-adjusted life years (DALYs) for 291 diseases and injuries in 21 regions, 1990–2010: a systematic analysis for the Global Burden of Disease Study 2010. The lancet, 2012. 380(9859): p. 2197–2223. doi: 10.1016/S0140-6736(12)61689-4 23245608

[6] Chamberlain D., et al., Trials of teaching methods in basic life support (3): Comparison of simulated CPR performance after first training and at 6 months, with a note on the value of re-training. Resuscitation, 2002. 53(2): p. 179–187. doi: 10.1016/s0300-9572(02)00025-4 12009222

[7] Rajeswaran L. and Ehlers V.J., Cardiopulmonary resuscitation knowledge and skills of registered nurses in Botswana. Curationis, 2014. 37: p. 1–7. doi: 10.4102/curationis.v37i1.1259 26852428

[8] F, B., Assessing the Need and Effect of Updating the Knowledge about Cardio-Pulmonary Resuscitation in Experts,. Journal of Clinical and Diagnostic Research., 2010. 4: p. 2512–2514.

[9] Nolan, J.P., et al., Resuscitation highlights in 2016. 2017, Elsevier. p. A1-A7. 10.1016/j.resuscitation.2017.02.001 28212838

[10] Neumar R.W., et al., Part 1: executive summary: 2015 American Heart Association guidelines update for cardiopulmonary resuscitation and emergency cardiovascular care. Circulation, 2015. 132(18_suppl_2): p. S315–S367. doi: 10.1161/CIR.0000000000000252 26472989

[11] Sreevastava D., et al., Cardio-pulmonary Resuscitation: an overview of Recent Advances in Concepts and Practices. Medical Journal Armed Forces India, 2004. 60(1): p. 52–58. doi: 10.1016/S0377-1237(04)80161-8 27407579 PMC4923515

[12] Atkins D.L., et al., Part 11: pediatric basic life support and cardiopulmonary resuscitation quality: 2015 American Heart Association guidelines update for cardiopulmonary resuscitation and emergency cardiovascular care. Circulation, 2015. 132(18_suppl_2): p. S519–S525. 26472999 10.1161/CIR.0000000000000265

[13] Manono B.K., Health Care Providers’ Knowledge, Skills and Institutional Factors that Determine Effective Cardiopulmonary Resuscitation at Nakuru County Hospital. 2022, JKUAT-COHES.

[14] Sintayehu Y., et al., Basic neonatal resuscitation skills of midwives and nurses in Eastern Ethiopia are not well retained: An observational study. PloS one, 2020. 15(7): p. e0236194. doi: 10.1371/journal.pone.0236194 32706775 PMC7380629

[15] Ssewante N., et al., Basic life support, a necessary inclusion in the medical curriculum: a cross-sectional survey of knowledge and attitude in Uganda. BMC Medical Education, 2022. 22(1): p. 140. doi: 10.1186/s12909-022-03206-z 35241065 PMC8892119

[16] Irfan B., et al., Current state of knowledge of basic life support in health professionals of the largest city in Pakistan: a cross-sectional study. BMC Health Services Research, 2019. 19(1): p. 865. doi: 10.1186/s12913-019-4676-y 31752855 PMC6868838

[17] Guteta M., Factors Affecting Cardiopulmonary Resuscitation Practice Among Nurses in Mizan Tepi University Teaching Hospital, Tepi General Hospital, and Gebretsadik Shawo Hospital, Southwest Ethiopia. Open Access Emerg Med, 2022. 14: p. 165–175. doi: 10.2147/OAEM.S350244 35462947 PMC9030545

[18] Mersha A.T., et al., Factors associated with knowledge and attitude towards adult cardiopulmonary resuscitation among healthcare professionals at the University of Gondar Comprehensive Specialized Hospital, Northwest Ethiopia: an institutional-based cross-sectional study. BMJ open, 2020. 10(9): p. e037416. doi: 10.1136/bmjopen-2020-037416 32988946 PMC7523201

[19] Liberati A., et al., The PRISMA statement for reporting systematic reviews and meta-analyses of studies that evaluate health care interventions: explanation and elaboration. Annals of internal medicine, 2009. 151(4): p. W-65-W-94. 10.7326/0003-4819-151-4-200908180-00136 19622512

[20] Chew KS H.F., Zarina Z, et al., A survey on the knowledge, attitude and confidence level of adult cardiopulmonary resuscitation among junior doctors in Hospital Universiti Sains Malaysia and Hospital Raja Perempuan Zainab II, Kota Bharu, Kelantan, Malaysia. Med J Malaysia., 2011. 66 (1). 23765145

[21] Bang A P.A., Bellad R, et al., Helping babies breathe (HBB) training: what happens to knowledge and skills over time?;16(1):364. BMC Pregnancy Childbirth., 2016. 16(1): p. 364. 27875999 10.1186/s12884-016-1141-3 PMC5120476

[22] Manono BK, M.A., Chakaya J., Assessment of knowledge and skills of cardiopulmonary resuscitation among health workers at Nakuru County Referral Hospital. Int J Community.. Med Public Health., 2021. 8: p. 3224–30.

[23] Munn Z., et al., Methodological guidance for systematic reviews of observational epidemiological studies reporting prevalence and cumulative incidence data. JBI Evidence Implementation, 2015. 13(3): p. 147–153. doi: 10.1097/XEB.0000000000000054 26317388

[24] Hoy D., et al., Assessing risk of bias in prevalence studies: modification of an existing tool and evidence of interrater agreement. Journal of clinical epidemiology, 2012. 65(9): p. 934–939. doi: 10.1016/j.jclinepi.2011.11.014 22742910

[25] Rücker G., et al., Undue reliance on I 2 in assessing heterogeneity may mislead. BMC medical research methodology, 2008. 8: p. 1–9. 19036172 10.1186/1471-2288-8-79 PMC2648991

[26] Green., J.P.H.a.S., Cochrane Handbook for Systematic Reviews of Interventions: Chapter 9: Analyzing data and undertaking meta-analyses, 9.5. Heterogeneity.. 2008.: p. 276–278.

[27] Sterne J.A. and Egger M., Funnel plots for detecting bias in meta-analysis: guidelines on choice of axis. Journal of clinical epidemiology, 2001. 54(10): p. 1046–1055. doi: 10.1016/s0895-4356(01)00377-8 11576817

[28] Egger M., et al., Bias in meta-analysis detected by a simple, graphical test. Bmj, 1997. 315(7109): p. 629–634. doi: 10.1136/bmj.315.7109.629 9310563 PMC2127453

[29] Gebreegziabher E., Aregawi A., and Getinet H., Knowledge and skills of neonatal resuscitation of health professionals at a university teaching hospital of Northwest Ethiopia. World journal of emergency medicine, 2014. 5(3): p. 196. doi: 10.5847/wjem.j.issn.1920-8642.2014.03.007 25225584 PMC4163816

[30] Tadesse M., et al., Knowledge, attitude, and practice towards basic life support among graduating class health science and medical students at Dilla University; a cross sectional study. Annals of Medicine and Surgery, 2022. 82: p. 104588. doi: 10.1016/j.amsu.2022.104588 36268360 PMC9577529

[31] SOLOMON, K., ASSESSMENT OF KNOWLEDGE, ATTITUDE AND ASSOCIATED FACTOR OF CARDIOPULMONARY RESUSCITATION AMONG RESIDENTS AT SAINT PAUL’S HOSPITAL MILLENNIUM MEDICAL COLLEGE, ADDIS ABABA, ETHIOPIA. 2021.

[32] Bedilu G.W., Eyayalem M.G., and Kidest G.M., Assessment of knowledge, attitude and associated factors of cardiopulmonary resuscitation among anesthetists working in governmental and private hospitals in Addis Ababa, Ethiopia: institutional based cross-sectional study. International Journal of Medicine and Medical Sciences, 2017. 9(3): p. 17–21.

[33] Kassie D.G. and Salih M.H., Study department and gender affects the knowledge and attitude of students towards cardio pulmonary resuscitation procedure at the University of Gondar, northwest Ethiopia, 2019. American Journal of Cardiovascular Disease, 2021. 11(4): p. 441. 34548941 PMC8449198

[34] Bogale M., et al., Assessment of knowledge of Nicu nurses and midwives in neonatal resuscitation in four urban hospitals in Addis Ababa, Ethiopia. Ethiopian Journal of Pediatrics and Child Health, 2021. 16(2).

[35] Kelkay M., et al., A cross sectional study on knowledge, practice and associated factors towards basic life support among nurses working in amhara region referral hospitals, northwest Ethiopia, 2016. Hos Pal Med Int Jnl [Internet], 2018. 2(2): p. 123–30.

[36] Bizuwork K., et al., The extent of Knowledge and practice toward neonatal resuscitation among nurses and midwives in public hospitals of South Wollo, northeast Ethiopia: Cross-sectional study. 2019.

[37] Abebe T.A., et al., Health-care providers’ knowledge, attitudes, and practices regarding adult cardiopulmonary resuscitation at Debre Markos Referral Hospital, Gojjam, Northwest Ethiopia. Advances in Medical Education and Practice, 2021: p. 647–654. doi: 10.2147/AMEP.S293648 34163280 PMC8214334

[38] Bekele F.A., Assimamaw N.T., and Ali M.S., Knowledge and associated factors towards neonatal resuscitation among nurses and midwives at the University of Gondar Comprehensive Specialized Hospital, Northwest Ethiopia. International Journal of Africa Nursing Sciences, 2021. 15: p. 100365.

[39] Mersha A., et al., Basic Newborn Resuscitation: Health Care Providers’ Level of Knowledge and Factors Affecting in the Hospitals of Southern Ethiopia. Journal of Neonatology, 2020. 34(4): p. 187–195.

[40] BIKAMO, E., KNOWLEDGE, ATTITUDE, PRACTICE, AND ASSOCIATED FACTORS TOWARDS BASIC LIFE SUPPORT AMONG NURSES WORKING AT ADULT EMERGENCY UNITS OF FEDERAL HOSPITALS, ADDIS ABABA, ETHIOPIA, 2021. 2021.

[41] Abebaw M., Tesfaye A., and Godie Y., Magnitude and associated factors of neonatal resuscitation among health care providers in selected public hospitals of Addis Ababa, Ethiopia, 2022. Multi center cross-sectional study.

[42] Benwu K.M., et al., A Cross Sectional Study on Knowledge and Practice Towards Cardiopulmonary Resuscitation Among Health Professionals Working at Mekelle University, Mekelle Ethiopia. 2021.

[43] Sintayehu Y., Knowledge of Basic Neonatal Resuscitation and Associated Factors Among Midwives and Nurses in Public Health Institutions in Eastern Ethiopia. Int J Gen Med, 2020. 13: p. 225–233. doi: 10.2147/IJGM.S255892 32547164 PMC7266389

[44] Irfan B., et al., Current state of knowledge of basic life support in health professionals of the largest city in Pakistan: a cross-sectional study. BMC health services research, 2019. 19: p. 1–7. 31752855 10.1186/s12913-019-4676-y PMC6868838

[45] Alkubati S.A., et al., Basic life support knowledge in a war-torn country: a survey of nurses in Yemen. BMC Nursing, 2022. 21(1): p. 141. doi: 10.1186/s12912-022-00923-0 35668520 PMC9169348

[46] Akinbodewa A., et al., Knowledge of Basic Life Support Among Doctors and Nurses Attending a Refresher Course in a Teaching Hospital in Southwest Nigeria: Knowledge of Basic Life Support. Nigerian Medical Journal, 2022. 63(4): p. 304–311. 10.60787/NMJ-63-4-77 PMC11163263 38863472

[47] Rajeswaran L., et al., Assessment of nurses’ cardiopulmonary resuscitation knowledge and skills within three district hospitals in Botswana. African Journal of Primary Health Care and Family Medicine, 2018. 10(1): p. 1–6. doi: 10.4102/phcfm.v10i1.1633 29781687 PMC5913783

[48] Sachdeva S., A study to assess knowledge andpractice of basic life support among nurses working in tertiary care hospital, New Delhi, India. Nurs Care Open Access J, 2020. 7(2): p. 48–52.

[49] Papi M., Hakim A., and Bahrami H., Relationship between knowledge and skill for basic life support in personnel of emergency medical services, Islamic Republic of Iran. Eastern Mediterranean Health Journal, 2020. 26(10): p. 1193–1199. doi: 10.26719/emhj.19.018 33103746

[50] HA Z. and SM S., Assessment of basic life support knowledge among nursing professionals. Egyptian Journal of Occupational Medicine, 2020. 44(1): p. 455–470.

[51] Munezero J.B.T., et al., Assessment of nurses knowledge and skills following cardiopulmonary resuscitation training at Mbarara Regional Referral Hospital, Uganda. Pan Afr Med J, 2018. 30: p. 108. doi: 10.11604/pamj.2018.30.108.15398 30364487 PMC6196081

[52] Ihunanya O.M., et al., Knowledge, attitude and practice of cardiopulmonary resuscitation among nurses in Babcock University Teaching Hospital in Ilishan-Remo, Ogun State, Nigeria. International Journal of Caring Sciences, 2020. 13(3): p. 1773–1782.

