# Supplementary material for: Health Professionals’ knowledge and practice on basic life support and its predicting factors in Ethiopia: Systematic review and meta-analysis
Source: PLoS One. 2024 Apr 9;19(4):e0297430. doi: 10.1371/journal.pone.0297430 (PMC11003682; doi:10.1371/journal.pone.0297430)
Supplement: Attachment — Submitted filename: review assignment.docx [file pone.0297430.s004.docx]

It is a great pleasure for me to get the opportunity to review a manuscript entitled ‘Health Professionals’ knowledge and Practice on Basic Life Support and its predicting factors in Ethiopia: Systematic Review and Meta-analysis’

I would like to appreciate the authors for their efforts go this far to investigate the impacts of professionals’ knowledge, practice and associated factors on Basic life support. However, I have some concerns that need to be address before providing my recommendation for publication.

***Title***

The tittle seems fine, but I personally don’t prefer the factor to be on the title as primary study rather you better include meta-regression for covariates and factor analysis could be described in data analysis section along with sub-group analysis and meta-regression.

Abstract:

**Background section:** it could incorporate the summary of the magnitude of BLS, discrepancies globally in global context, and justification of your review in 3-4 lines.

**Method:** A comprehensive search strategy was conducted in…….. From ……to….with/without language restrictions or without date and language restriction; No database called international or you may use big databases if you like to say so.

Conclusion: ‘how low is considerably low?’

***Introduction***

I am sorry to say but this section is poorly written part of the manuscript. It lacks components that needs to be addressed in this section which might include but not limited to description of the problem, risk factors, etiology, impacts, outcomes

*There are plenty of questions that need to be addressed:*

- What are the magnitude of cardiopulmonary arrest, and other problems that could be addressed with basic life support from global to local context?
- What are gaps you identified during your literature search?
- Why did you pan this review?
- What is the prevalence of low knowledge level of BLS among health professionals from the available literatures globally?
- What is the prevalence of low practical skill of BLS among health professionals from the available literatures globally?
- With whom these skills are very low the most?

***Method***

The method section subheadings should be organized as Protocol and registration, Eligibility criteria, Comprehensive search, Selection of studies, Critical appraisal, Data extraction…….

The search should be comprehensive and you have to report a complete comprehensive search strategy at least for one database. Where and how did you get the Mesh terms for PubMed and other databases?? How knowledge and skill was measured in the primary studies?? How valid the cut point was?

***Data-analysis***

Subgroup analysis could be conducted by categorical moderators such as health care provider, year of experience, study setting etc. and meta-regression on covariates such as mean age, experience, sample size, quality score, and others. Besides, factor analysis better be conducted by age, profession, experience, gender, and other moderators

***Result***

The result lacks comprehensiveness where it could include the summary of search result, and description of the included studies in terms of mean age, sample size, profession, study setting, and how these studies were addressed specifically for practice assessment which might have biases.

**Meta-analysis**

The met-analysis better could be done with metaprop command rather than drop down menu as it has a wide margin of safety. The factor analysis effect estimate should be reported with AOR with 95% CI on the forest plot

***Discussion***

The discussion is very shallow with limited comparison and impact analysis of your finding with the available literatures.

The discussion should be finalized with the following subheadings

- Limitation and strength
- Implication for policy-makers
- Implication for clinical practice
- Implication for further studies

***Generally, I would say the topic is very relevant, but the authors have a lot to do.***
